# Barriers and Facilitators for Implementing Interventions for Treating Patients With Chronic Musculoskeletal Pain: A Qualitative Scoping Review Using the Theoretical Domains Framework

**DOI:** 10.1002/msc.70108

**Published:** 2025-04-26

**Authors:** Mette Dahl Klausen, Line Lindberg, Simon Kristoffer Johansen, Michael Skovdal Rathleff, Kristian Damgaard Lyng

**Affiliations:** ^1^ Department of Health Science and Technology Faculty of Medicine Aalborg University Aalborg Denmark; ^2^ The National Research Centre for the Working Environment The Ministry of Employment Copenhagen Denmark; ^3^ Department of Clinical Medicine Center for General Practice at Aalborg University Aalborg University Aalborg Denmark

**Keywords:** barriers and facilitators, implementation science, implementation strategy, musculoskeletal pain, qualitative research, scoping review, theoretical domains framework

## Abstract

**Background:**

Chronic musculoskeletal (MSK) pain poses a significant societal burden, yet many evidence‐based interventions fail to reach clinical practice, highlighting an implementation gap. This study aimed to identify barriers and facilitators in implementing MSK pain interventions across healthcare settings.

**Methods:**

We conducted a scoping review following the Joanna Briggs Institute approach. We searched MEDLINE, EMBASE, CINAHL, PubMed and PsycINFO (up to December 2023) for peer‐reviewed qualitative studies exploring the perspectives of patients (18+) and healthcare professionals (HCPs) on implementing MSK pain interventions. Studies were screened independently by two authors, and data were analysed using the General Inductive Approach and mapped to the Theoretical Domains Framework (TDF).

**Results:**

From 18,220 records, 22 studies were included, involving 307 HCPs and 76 patients across all healthcare sectors. Six major themes emerged: (1) long way from usual care, (2) trust and commitment, (3) support, (4) time and finance, (5) knowledge and skills and (6) patient preferences. The most frequently reported TDF domain was ‘Environmental Context and Resources’, with key barriers including time constraints, low reimbursement, and insufficient support. Facilitators included leadership, communication, and social networks.

**Conclusion:**

Implementation of MSK pain interventions is hindered by systemic barriers at individual, organisational, and political levels. Addressing these barriers through targeted strategies is essential for improving sustainable clinical implementation and enhancing patient care.

## 1 Background

Chronic musculoskeletal (MSK) pain is the leading cause of disability worldwide (James et al. 2018). Chronic MSK pain has extensive socioeconomic consequences, including direct healthcare costs of treating patients and indirect costs such as lost workplace productivity (Chen et al. 2023; Gorasso et al. 2023). The socioeconomic costs of chronic pain are estimated to run into billions in Europe annually (Stubhaug et al. 2024). Furthermore, data from the US show that the socioeconomic costs exceed the combined costs of cardiovascular disease and cancer (Breivik et al. 2013; Gaskin and Richard 2012). While recent studies highlight that chronic MSK pain is on the rise and despite large efforts from experts, little progress has been made to reduce the burden of chronic MSK pain (Safiri et al. 2021). Despite the growing body of knowledge on treatments for managing chronic MSK pain, research has shown that most interventions do not reach clinical practice and approximately 40% of these patients do not receive evidence‐based interventions (Landhuis 2016). This highlights the existence of an implementation gap that warrants further investigation. Several factors might influence the implementation process at the individual, organisational, and political levels (Ernstzen and Louw 2024). On an organisational and political level, policy, regulation, and finance models (which may differ within each healthcare sector) influence the process (Briggs et al. 2019). Individual factors are likely to affect the implementation, for example, when healthcare professionals (HCPs) and patients maintain outdated beliefs and attitudes (Briggs et al. 2019; Setchell et al. 2017). Implementation is complex and challenging due to the involvement of various stakeholders on different organisational levels regarding different individuals and their interacting relations in different healthcare sectors (May et al. 2016). Researchers and clinicians are often forced to plan, develop or select implementation strategies with little information about what might work and little consideration of potential barriers and facilitators (Fernandez et al. 2019). To ensure long‐term, sustainable implementation of interventions for patients living with chronic MSK pain, there is a need to understand the barriers and facilitators better when implementing interventions. Therefore, this scoping review aims to identify perceived patient and HCP barriers and facilitators when implementing interventions for managing patients with chronic MSK pain across different healthcare settings.

## 2 Methods

To identify barriers and facilitators for the implementation of interventions in the management of people with chronic MSK pain, we applied a scoping review approach in accordance with the approach defined by the Joanna Briggs Institute (JBI) (M. D. J. Peters et al. 2015, 2020). The JBI method from the Joanna Briggs Institute involves a systematic approach to scoping reviews to map evidence related to a specific research question using transparency and rigour in the selection and analysis of relevant literature (M. D. J. Peters et al. 2015, 2020). In addition to the review approach, we used an inductive approach to code and thematise data followed by mapping identified factors into TDF for an overview of the distribution within the TDF domains. The protocol for this review was pre‐registered and uploaded to the Open Science Framework (https://doi.org/10.17605/OSF.IO/83AZD). The reporting of the scoping review followed the Preferred Reporting Items for Systematic reviews and Meta‐Analyses extension for Scoping Reviews (PRISMA‐ScR) (Tricco et al. 2018).

### 2.1 Eligibility Criteria

This scoping review considered all types of qualitative studies exploring the perspectives of implementing interventions from patients with chronic MSK pain, and HCPs working with the patient population. In this study, ‘intervention’ is a treatment, procedure, or other action taken to prevent or treat the patient's condition or improve their health in different ways. We defined ‘implementation’ as methods to promote the systematic uptake of research findings and other evidence‐based practices into routine practice, and, hence, to improve the quality and effectiveness of health services. We included studies conducted in any healthcare setting in any country. The study age limit applied to the literature search was 20 years, and participants' age was set to include 18+ years. Studies were excluded if they explored experiences of palliative care or treating cancer pain, postoperative pain, pregnancy‐related low‐back pain, or prescription opioids or drugs in MSK pain care. All quantitative studies and studies in languages other than English were excluded.

### 2.2 Information Sources and Search Strategy

The systematic literature search was designed to identify articles related to the existing barriers and facilitators to implementing interventions in any healthcare setting for patients with chronic MSK pain. We tailored the systematic literature search to the following databases: PubMed, Embase, Scopus, CINAHL, and PsycINFO. Medical subjects' headings and text related to ‘Chronic musculoskeletal pain’, ‘Implementation’, and ‘Qualitative research’ were used. We designed the search strategy in collaboration with an experienced research librarian. The entire literature search is presented in Supporting Information S1: Appendix 1.

### 2.3 Screening and Selection

Search results were imported and collated into Covidence Systematic Review Software (Veritas Health Innovation, Melbourne, Australia) to manage all records and remove duplicates. Three authors (M.D.K., L.L., K.D.L.) independently screened titles and abstracts. At least two authors independently read 20% of the full texts to identify studies that met the inclusion criteria before one author did the remaining full text readings. Any concerns were regularly discussed within the author group until consensus was reached. Corresponding authors were not contacted for further clarity or additional data.

### 2.4 Data Charting Process

Data were extracted from the included studies by two authors using Covidence using a pre‐designed charting form. Two authors independently extracted data (M.D.K., L.L.) and held several consensus meetings to discuss potential disagreements. In case of doubt in this phase, a third author was consulted (K.D.L.) in case of conflicts or for double‐checking the final decision. The following data were extracted: author name(s), year of publication, country, participants' characteristics and sample size, healthcare setting, intervention, methods of implementation, methods of evaluation, authors' main conclusion and comments on limitations of methods.

### 2.5 Data Synthesis and Presentation of Results

The data analysis was conducted in a two‐step process, which included the identification and synthesis of recurring and salient themes present within the included studies (inductive analysis), and a secondary interpretation via the Theoretical Domains Framework. Firstly, we used the general inductive approach described by Thomas as a framework for systematically identifying, categorising, and synthesising qualitative insights from HCPs and patients with chronic MSK pain within all included studies (Thomas 2006). The general inductive analysis was chosen based on its orientation towards identifying underlying patterns of experiences and processes across multiple datasets (Thomas 2006). The data analysis followed the five‐step approach outlined by Thomas (2006). At first, the authors independently coded raw data using NVivo software hosted by (Aalborg University) (QSR International Pty Ltd., version R1/2020, NVivo). The analysis was guided by the evaluation objectives, ‘barriers and facilitators’ which informed the identification of thematic categories, sub‐categories, and their interacting relationships. When the authors of the original studies did not communicate their findings as barriers or facilitators, we assessed whether the data influenced the results positively (facilitator) or negatively (barrier) to ensure that the emerging themes or sub‐themes were correctly linked to the analysis objectives. Before commencing the coding, we sample‐coded two articles to validate the evaluation objectives. During the analysis, disputes about coded themes were resolved by discussion until a consensus was reached. Secondly, we selected the Theoretical Domains Framework (TDF) as a framework to explore the extracted barriers and facilitators (cognitive, affective, social, and environmental) that influence behaviour with a theoretical lens (Atkins et al. 2017). Three authors independently mapped at least 20% of the barriers and facilitators into TDF domains and agreed on more than 90%; the remaining were done by two authors. Any deputies were resolved by discussion until an agreement was reached. Individual barriers and facilitators were coded separately and subsequently quantified, with counts and percentages reported for each TDF domain.

## 3 Results

### 3.1 Study Selection

**FIGURE 1:**
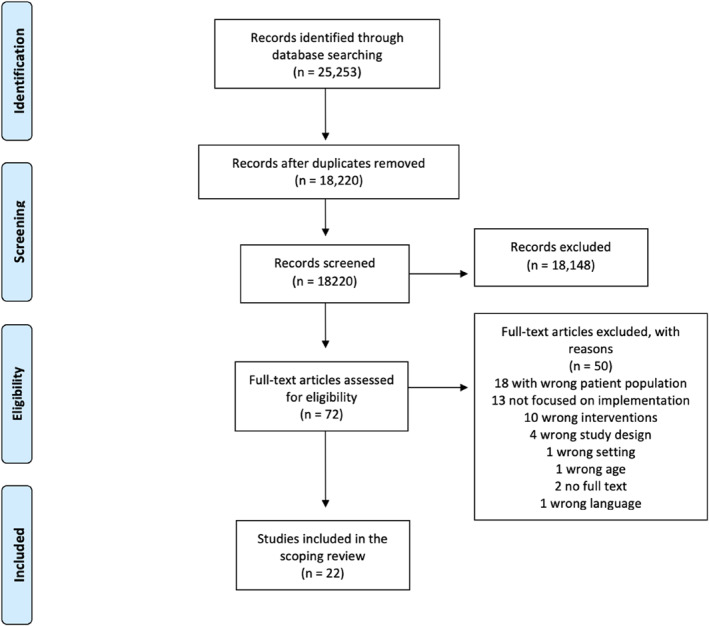
PRISMA flowchart.

The selection of studies is presented in a flowchart (Figure 1). Our search yielded 25,253 citations. A total of 18,220 individual studies were screened based on titles and abstracts, of which 72 were included for full‐text reading. The total number of included studies for data extraction was 22 studies (total *n* = 307 HCPs, *n* = 76 patients), and all included studies were published between a period of 2013–2022 (Barker et al. 2016; Bøgdal et al. 2021; Bouma et al. 2022; Brewer et al. 2021; Bunzli et al. 2016; Cowell et al. 2018, 2019; Cuperus et al. 2013; Faymonville et al. 2021; Fritz et al. 2019; Holopainen, Piirainen, et al. 2022; Holopainen, Vuoskoski, et al. 2022; Kaloty et al. 2023; Lentz et al. 2023; Littlewood et al. 2015; S. Peters et al. 2016; Richmond et al. 2018; Spitaels et al. 2017; Stenberg et al. 2017; Synnott et al. 2016; van der Vaart et al. 2019; Waddington et al. 2017).

### 3.2 Characteristics of Included Studies

Most of the included studies (*n* = 16) explored the perspective of HCPs, seven studies explored patient perspectives (*n* = 7), and one study examined both perspectives. Chronic low back pain (*n* = 10) and unspecified chronic pain (*n* = 8) were the most common pain conditions targeted, followed by hip or knee osteoarthrosis (*n* = 3). Of the included studies, 14 were conducted in primary care, two in secondary care, four in tertiary care, and two were multisector studies (i.e., combined primary and secondary care and all sectors). Eleven countries were represented across the included studies, including the United Kingdom (*n* = 5), Netherlands (*n* = 3), Canada (*n* = 2), Australia (*n* = 1), Ireland (*n* = 2), Denmark (*n* = 2), Sweden (*n* = 2), Finland (*n* = 2), the United States (*n* = 2), Belgium (*n* = 1), and Germany (*n* = 1). Most interventions followed a biopsychosocial approach, which considers biological, psychological, and social factors and their complex interactions in understanding health, illness, and healthcare delivery (42, 43), except for two studies that evaluated specific tools (i.e., a booklet and virtual delivery). An overview of the included studies is presented in Table 1.

**TABLE 1:** Overview of included studies.

Authors (year), country	Participants	Setting	Intervention	Methods of implementation	Methods of evaluation	Authors' main conclusions	Comments
Barker et al. (2016), UK	Physiotherapists (*n* = 7)	Secondary care	Acceptance and commitment therapy (ACT) for patients with chronic MSK pain	Training and mentoring in ACT from expert practitioners through role‐play and experiential learning	Ad hoc reflective sessions in focus groups, and diaries	Overall, the physiotherapist recognised a positive progression in pain management, and one physio felt comfortable with the skill set with other psychological‐based tools used by physiotherapists	Discrepancy between aim and conclusion Unclarity regarding participants (a clinical lead and an assistant practitioner)
Bouma et al. (2022), NL	HCP (*n* = 38)	Primary and secondary care	Lifestyle interventions (physical activity and/or eating behaviour) in patients with OA	All methods that HCPs can implement to promote a healthy lifestyle or influence patients' physical activity and/or eating behaviour	Four focus groups with semi‐structured interview guides	The implementation of lifestyle interventions is affected by both individual and environmental factors. Interdisciplinary collaboration is important, but other factors vary widely	
Brewer et al. (2021), CND	Physiotherapist (*n* = 11)	Primary care	ChrOnic pain self‐ManageMent support with pain science EducatioN and exerCisE	Physiotherapists trained in the components of delivery and receive feedback	Semi‐structured telephone interviews	The study provides an understanding of experience, barriers, facilitators, benefits, and drawbacks. Pain‐management programs for chronic pain can be implemented in primary care	
Bunzli et al. (2016), AUS and IRL	Patients with chronic LBP (AUS *n* = 5, IRL *n* = 9)	Primary care	Cognitive functional therapy (CFT)	Compare the perspectives of participants who reported differing levels of improvement after CFT	Semi‐structured interviews	Patients adopting the intervention diverge. A successful outcome dependent on the biopsychosocial pain beliefs and independence among participants. Ongoing support is needed in small improvers	Authors provide professional development workshops on CFT for clinicians
Bøgdal et al. (2021), DK	Patients with chronic LBP (*n* = 9)	Tertiary care	Integrated multidisciplinary rehabilitation programme	Combination of inpatient and home‐based activities	Semi‐structured interviews	Patients believed the knowledge was beneficial and meaningful when based on individual needs and preferences. The patients integrated very well the knowledge, skills and behaviours into their everyday life	The patients were recruited from one highly specialised rheumatic rehabilitation centre in Denmark
Cowell et al. (2018), UK	Physiotherapists (*n* = 10)	Primary care	CFT	Education in implementation of CFT	Semi‐structured interviews	Physiotherapists value the treatment but felt underprepared in knowledge and time to address cognitive and emotional factors	Authors provide professional development workshops on CFT for clinicians Same interviews seem to have been used for two different projects
Cowell et al. (2018), UK	Physiotherapists (*n* = 10)	Primary care	CFT	The 10‐month programme included educator‐led training, and problem‐based learning, followed by 6‐month clinical mentoring	Semi‐structured interviews	Physiotherapists self‐reported confidence and competence enhanced, ongoing support and clinical integration should be included	Authors provide professional development workshops on CFT for clinicians Same interviews seem to have been used for two different projects
Cuperus et al. (2013), NL	Patients with hip or knee OA (*n* = 17)	Primary care	Self‐management booklet	Patients received the booklet from their general practitioner or from researchers and were instructed on how to use it	Interviews with open‐ended questions	Patients legitimise non‐use of the booklet by the lack of encouragement given by their HCPs and by their perceived doubts concerning the HCPs' endorsement of non‐surgical treatment for OA	Only 4/17 are booklet users
Faymonville et al. (2021), DK	HCPs, administrative staff, kitchen and nutrition staff, housekeeping and property managers and management (*n* = 31)	Tertiary care	Integrated multidisciplinary rehabilitation programme	Implementation via enrolment in a randomised controlled trial	Four focus groups with semi‐structured interview guide	Implementation challenges professional competence and role. All different staff should be involved in implementation, as participation contributes to increased positivity in relation to new initiatives	The intervention is both in‐patient care and home‐based activity
Fritz et al. (2019), S	Physiotherapists, patients with chronic pain and managers (*n* = 11)	Primary care	A behavioural medicine approach	Implementation of change model	Semi‐structured interviews through video recorded treatment sessions and documents with local directives	Education alone is not sufficient to implement intervention. Most frequently determinants regarding the PT and the patient, but also the treatment itself. Professional interaction resources seem also important	
Holopainen et al. (2020), FI	Physiotherapists (*n* = 22)	Primary care	CFT	4–6 days workshops with lectures, group discussions and patient demonstrations	Semi‐structured interviews	Physiotherapists' conceptions of implementation varied greatly. For some, the training was insufficient to support adequate changes in their practice behaviour and that for others it was motivated and a life changing experience	Authors provide professional development workshops on CFT for clinicians
Holopainen et al. (2020), FI	Patients with persistent LBP (*n* = 10)	Primary care	CFT	Physiotherapists with 4–6 days CFT training, without mentoring or clinical observation	Semi‐structured recall interviews utilising the participants' previously videotaped initial sessions on average after 1.5 years	The patients' conceptions varied. Some felt disappointed and abandoned by the healthcare system, did not become independent in self‐management, and felt stigmatised and dependent on others. Others felt supported to understand, make sense of pain, and learnt new skills to take control	Authors provide professional development workshops on CFT for clinicians
Kaloty et al. (2023), CND	Patients with chronic pain (*n* = 15)	Tertiary care	Virtual delivery of exercise interventions	Due to the COVID‐19 pandemic, the clinic was challenged and therefore they used the virtual care as an interdisciplinary chronic pain management programme	Semi‐structured interviews by telephone	Virtual care can supplement in‐person care and improve access. The impact of technology, the home environment, pain, supervision, and feedback could be facilitators or barriers. Tailored care, preparation and additional support is needed	Most participants indicated that they were comfortable using technology, few were neutral, but none were uncomfortable
Lentz et al. (2023), US	All stakeholders involved in programs from public and private payers, researchers, and policymakers (*n* = 53)	Multisector	Integrated pain management programme for chronic pain (e.g., nutrition, behavioural health, social services, legal aid)	All stakeholders involved in programs that deliver biopsychosocial care within a structured programme	Semi‐structured interviews tailored to different stakeholders, and four original case studies	Integrated biopsychosocial programs (nonpharmacological approaches) are beneficial to manage MSK pain, but these programs are not widely implemented	
Littlewood et al. (2015), UK	Physiotherapists (*n* = 13)	Secondary care	Self‐managed exercise intervention in patients with rotator cuff tendinopathy	2‐h training sessions led by the first author. Participants were offered follow‐up appointments as required to facilitate	Semi‐structured interviews	Clear differences between the new self‐management exercise intervention and their preferred approach. For the intervention differed to such an extent that implementation would be challenging	The first author delivered the intervention
S. Peters et al. (2016), DE	Physicians, psychologists, physiotherapists, exercise therapists and occupational therapists (*n* = 45)	Primary care	Interdisciplinary, standardised curriculum back schools (CBS)	Two different implementation interventions: Train‐the‐trainer workshops and a written implementation guide	Semi‐structured interviews 12 weeks after the implementation of the CBS had been initiated	This study covered barriers and facilitators of the implementation. Results are explorative and hypothesis generating and provide potential explanatory mechanisms for behaviour and acceptance of HCPs in implementation	
Richmond et al. (2018), UK	Physiotherapists (*n* = 11)	Primary care	Best skills training. within groups: Cognitive behavioural approach (CBA) combined with exercise to LBP	Face‐to‐face training and 10‐h online learning. A manual with session‐by‐session plans	Semi‐structured interviews	CBA is beneficial and might be implemented. Implementation strategies targeted behaviour change identified in this study may be appropriate for managing patients with LBP	
Spitaels et al. (2017), BE	Patients with knee OA (*n* = 11)	Primary care	Clinical guidelines	A guideline	Semi‐structured interviews	Barriers are related to both the patients and healthcare professionals and can cause nonadherence. These identified barriers and facilitators should be considered in future	
Stenberg et al. (2017), S	HCPs (*n* = 14)	Primary care	Multimodal pain rehabilitation (MMR)	The structure of the team and the offered MMR programme could differ from centre to centre, and depending on the patient's needs	Individual interviews with open‐ended questions	Managers at all organisational levels must take responsibility and priority to facilitate the implementation of MMR in primary care. A driving HCP is a facilitator initial. The whole team and good teamwork is important	Each MMR unit included was offered financial incentives as a government strategy to implementing
Synnott et al. (2016), IRL	Physiotherapists (*n* = 13)	Primary care	CFT	CFT training with workshop attendance and supervision of clinical practice	Semi‐structured telephone and Skype interviews	After CFT training physiotherapists described increased confidence and skills to manage biopsychosocial factors. They also reported increased confidence and job satisfaction because of addressing cognitive, psychological, and social factors	Some of the authors are the founders of the intervention used in this study
van der Vaart et al. (2019), NL	HCPs and team managers (*n* = 18)	Primary care	Cognitive behavioural therapy via internet (ICBT) as an additional support tool to combine with other protocols	Agreement with managers with respect to the use of the treatment, and capacity. Afterwards, therapist training and supervision with monthly contact	Semi‐structured interviews	Barriers and facilitators for implementing ICBT are identified from healthcare professionals and managers, which could be taken into consideration in broader implementation projects	The included therapist worked with either chronic pain or chronic fatigue, and data is presented pooled
Waddington et al. (2017), US	HCPs (*n* = 13)	Tertiary care	Combined self‐management and yoga	The patient tried implementing yoga to establish self‐management in a pain management clinic	Semi‐structured interviews (seven individual interviews and one focus group interview)	Yoga paired with self‐management is feasible to implement to address chronic pain in a clinical setting. It is facilitating when all staff accept and believe in the intervention. Furthermore, the staff‐patient relationship is important	Not clear if all interviewees were HCPs (yoga instructor background is unclear)

### 3.3 Initial Coding

The initial coding resulted in a total of 381 codes, which we reduced to 229 by removing repeated codes, and codes that could not be accommodated within the most frequent, dominant, or significant themes inherent in raw data. Patients' perspective represented 67 codes (29%), and 162 (71%) codes from the HCP's perspective, for an overview of the codes referred to see Supporting Information S1: Appendix 2.

### 3.4 Identifying Themes From Data

**FIGURE 2:**
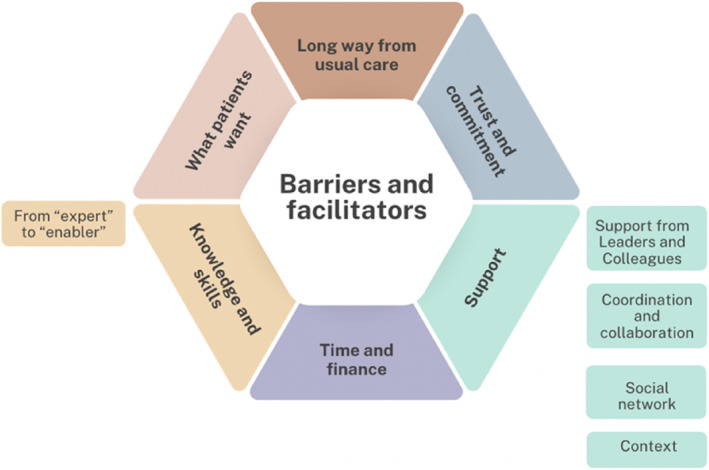
Overview of major themes and subthemes.

The 229 codes were merged and reorganised into six major themes and six subthemes (Figure 2). The major themes were formulated as ‘Long way from usual care’, ‘Trust and commitment’, ‘Support’, ‘Time and finance’, ‘Knowledge and skills’ and ‘What patients want’.

### 3.5 Long Way From Usual Care

A major barrier to the implementation of interventions was the perception of interventions being ‘long way from usual care’ for both HCPs and patients (Barker et al. 2016; Faymonville et al. 2021; Holopainen, Piirainen, et al. 2022; Richmond et al. 2018; Synnott et al. 2016). Three studies described how HCPs felt the implementation challenged their professional role (Barker et al. 2016; Faymonville et al. 2021; Richmond et al. 2018). This made them feel uncomfortable (Barker et al. 2016; Faymonville et al. 2021; Richmond et al. 2018), that they had to compromise their therapeutic alliance with the patients (Barker et al. 2016; Faymonville et al. 2021; Richmond et al. 2018), and felt like they failed to ‘*fix*’ the patient (Barker et al. 2016; Faymonville et al. 2021; Richmond et al. 2018). Resistance to new interventions often arose when HCPs felt isolated within their work communities (Barker et al. 2016; Faymonville et al. 2021; Holopainen, Piirainen, et al. 2022; Richmond et al. 2018; Synnott et al. 2016), and the intervention contrasted with their usual care, which made the HCPs feel anxious and was tied to their concerns about not meeting patients' expectations of hands‐on interventions (Barker et al. 2016; Faymonville et al. 2021; Holopainen, Piirainen, et al. 2022; Richmond et al. 2018; Synnott et al. 2016). Furthermore, new interventions, which differed radically from usual care, may lead to opening a ‘can of worms’, which was a barrier because HCPs felt they did not have the professional capacity and skills to fully comprehend the intervention (Barker et al. 2016; Faymonville et al. 2021; Holopainen, Piirainen, et al. 2022; Richmond et al. 2018; Synnott et al. 2016). Hence, the HCPs seemed compelled to reconsider their practices, potentially indicating the necessity for organisational adjustments to accommodate these new approaches (Brewer et al. 2021; Holopainen, Piirainen, et al. 2022; Lentz et al. 2023; Stenberg et al. 2017; van der Vaart et al. 2019; Waddington et al. 2017).

### 3.6 Trust and Commitment

Trust and commitment played an important role when implementing new interventions (Bouma et al. 2022; Cuperus et al. 2013; Faymonville et al. 2021; Fritz et al. 2019; Holopainen, Piirainen, et al. 2022; Holopainen, Vuoskoski, et al. 2022; Richmond et al. 2018; Spitaels et al. 2017; Waddington et al. 2017). Committing to new interventions often hinges on trust, especially for patients who must believe in its effectiveness (Holopainen, Piirainen, et al. 2022). Negative beliefs about interventions pose as a barrier for both HCPs and patients (Bouma et al. 2022; Bunzli et al. 2016; Holopainen, Vuoskoski, et al. 2022; Waddington et al. 2017), as they may doubt (lack trust on) its benefits interventions (Bouma et al. 2022; Cuperus et al. 2013; Faymonville et al. 2021; Fritz et al. 2019; Holopainen, Piirainen, et al. 2022; Holopainen, Vuoskoski, et al. 2022; Richmond et al. 2018; Spitaels et al. 2017; Waddington et al. 2017). These doubts stem from previous adverse effects, lack of knowledge, or unmet patient expectations (Fritz et al. 2019; Holopainen, Vuoskoski, et al. 2022). While increased knowledge through patient demonstration facilitates implementation, it can become a barrier if it fosters a ‘Guru culture’, which may lead to blind trust in an authoritative person or a singular treatment (Holopainen, Piirainen, et al. 2022). Junior faculty members exhibited greater receptiveness to new interventions compared with HCPs in later career stages (Lentz et al. 2023; Littlewood et al. 2015). High‐quality research is deemed imperative for HCP commitment; however, some patients with OA were sceptical of the research, preferring to experience it for themselves and attached greater importance to information from their social network than from their HCPs (Brewer et al. 2021; Cowell et al. 2018; Holopainen, Piirainen, et al. 2022; Richmond et al. 2018; Spitaels et al. 2017; Waddington et al. 2017).

### 3.7 Support

Support was highlighted as an essential facilitator for successful implementation from both the patients and HCPs perspectives (Bouma et al. 2022; Brewer et al. 2021; Faymonville et al. 2021; Fritz et al. 2019; Holopainen, Piirainen, et al. 2022; Holopainen, Vuoskoski, et al. 2022; Spitaels et al. 2017; Stenberg et al. 2017; van der Vaart et al. 2019; Waddington et al. 2017). Support from leaders and colleagues, effective communication, social networks, and consideration of contextual factors were identified as important elements for the successful implementation of new interventions for patients with chronic MSK pain (Bouma et al. 2022; Brewer et al. 2021; Faymonville et al. 2021; Fritz et al. 2019; Stenberg et al. 2017; van der Vaart et al. 2019; Waddington et al. 2017).

#### 3.7.1 Subtheme 1—Support From Leaders and Colleagues

Some studies demonstrated that supportive leadership, colleagues with a similar understanding, and the opportunity to share clinical experiences were crucial facilitators for HCPs to commit to new interventions (Bouma et al. 2022; Brewer et al. 2021; Faymonville et al. 2021; Fritz et al. 2019; Stenberg et al. 2017; van der Vaart et al. 2019; Waddington et al. 2017). Importantly, implementation was further facilitated when HCPs experienced, they had the appropriate amount of time to prepare, deliver and discuss their challenges with their colleagues (Bouma et al. 2022; Brewer et al. 2021; Cowell et al. 2019; Faymonville et al. 2021; Fritz et al. 2019; Holopainen, Piirainen, et al. 2022; Lentz et al. 2023; Richmond et al. 2018). The low status of managing chronic MSK conditions compared with other conditions was described as a barrier to the implementation of new interventions (Stenberg et al. 2017). In relation to this, two studies identified that the HCPs didn't feel supported by the organisation and their colleagues not working with the new intervention (Holopainen, Piirainen, et al. 2022; Stenberg et al. 2017). Continuous external support was valued and a facilitator for the HCPs but could also serve as a barrier if the HCP became too dependent and could not solve simple questions or problems on their own (Holopainen, Piirainen, et al. 2022). Patients identified a lack of support and recognition from their employers as a barrier, exemplified by the absence of alternative work arrangements or adjustments to workload due to their condition (Holopainen, Vuoskoski, et al. 2022; Spitaels et al. 2017).

#### 3.7.2 Subtheme 2—Coordination and Collaboration

Communication was described as a facilitator to the implementation of new interventions for HCPs (Cowell et al. 2018; Holopainen, Piirainen, et al. 2022; van der Vaart et al. 2019). Some patients also highlighted connections between effective communication and a trusting relationship, which can facilitate accepting new interventions (Bunzli et al. 2016; Spitaels et al. 2017). Studies mentioned the importance of the HCPs speaking a common language to avoid patients receiving conflicting information from different HCPs (e.g., different interpretations of MRI results) (Bouma et al. 2022; Holopainen, Piirainen, et al. 2022). ‘A driving spirit’ (i.e., a passionate HCP responsible for implementation) within the HCP community may facilitate the implementation of new interventions, through both being a key facilitator for institutional (e.g., inspiring and supporting colleagues) and infrastructural (e.g., highlighting the importance to decision‐makers) changes (Bouma et al. 2022; Lentz et al. 2023; Stenberg et al. 2017; Waddington et al. 2017).

#### 3.7.3 Subtheme 3—Social Network

From the patients' viewpoint, a social support network was a facilitator to keep them motivated to comply with new interventions (Holopainen, Vuoskoski, et al. 2022; Spitaels et al. 2017). A barrier arose when patients did not receive empathy from their partner or family, and hence, a facilitator was identified in terms of establishing support networks from group interventions, which seemed to benefit some patients (Bøgdal et al. 2021; Holopainen, Vuoskoski, et al. 2022; Lentz et al. 2023; Waddington et al. 2017). Group interventions served as opportunities to share experiences and meet people in the same situation, which could embrace their openness, participation, and self‐management on a higher level (Holopainen, Vuoskoski, et al. 2022; Lentz et al. 2023). Programme administrators highlighted that group sessions might further scale up financially (Lentz et al. 2023; van der Vaart et al. 2019). Patients receiving a virtual intervention reported less supervision in group sessions versus individual sessions, and they described the need for an individualised consultation (Kaloty et al. 2023).

#### 3.7.4 Subtheme 4—Context

The environmental context may also influence the successful implementation of new interventions (Bouma et al. 2022; Lentz et al. 2023; Stenberg et al. 2017; Waddington et al. 2017). Physical co‐location of services and providers could facilitate regular communication and cross‐disciplinary discussions and make care more accessible (Bouma et al. 2022; Lentz et al. 2023). An organisational challenge was the lack of appropriately trained staff (Waddington et al. 2017), whereas HCPs reported that a limited health records system was a barrier to implementing new interventions (Bouma et al. 2022). Along the same line, patients who experienced difficulties in terms of navigating websites, and booking appointments were a barrier to the implementation of new interventions into their lives (Kaloty et al. 2023).

### 3.8 Time and Finance

Time was frequently reported as a barrier to implementing interventions (Bouma et al. 2022; Brewer et al. 2021; Cowell et al. 2019; Cuperus et al. 2013; Faymonville et al. 2021; Fritz et al. 2019; Holopainen, Piirainen, et al. 2022; Lentz et al. 2023; S. Peters et al. 2016; Richmond et al. 2018; Spitaels et al. 2017; van der Vaart et al. 2019). New interventions were more time‐consuming for administration, preparation, appointment time, and the number of consultations. One study explained that HCPs felt their professionalism was compromised, arguing with lack of time (Faymonville et al. 2021). Lack of time was also a barrier experienced by patients, resulting in a lack of information and limited encouragement from the HCPs (Cuperus et al. 2013). In addition, patients mentioned time as a barrier to performing physical activity (Spitaels et al. 2017). In general, low reimbursement rates are a barrier to the implementation (Bouma et al. 2022; Fritz et al. 2019; Lentz et al. 2023; Spitaels et al. 2017; Stenberg et al. 2017; van der Vaart et al. 2019; Waddington et al. 2017). For leaders, the return on investment facilitates buy‐in to build and sustain interventions (Lentz et al. 2023; Waddington et al. 2017). The downside of some reimbursement models was that the number of patient visits could become more important than the quality of the care (Fritz et al. 2019; Stenberg et al. 2017). A barrier for HCPs to deliver the intervention was limited coverage for certain interventions (e.g., physiotherapy) compared to the well‐reimbursement of surgical and pharmacological treatments (Bouma et al. 2022; Lentz et al. 2023). Moreover, those financings are less likely to provide reimbursement to patients if they are not familiar with the type of intervention the patients receive (Lentz et al. 2023; van der Vaart et al. 2019). Political and organisational factors may also affect the HCP's receptivity to changes in behaviour to new interventions (Bouma et al. 2022; Fritz et al. 2019; Lentz et al. 2023; Stenberg et al. 2017). In one study, the HCPs expressed they wanted a more structured approach from the political or organisation, and not just leaving it up to the individual HCP (Stenberg et al. 2017). Patients felt the healthcare system failed to support them with their ongoing financial problems and disability, meaning they had to depend on their partners and rehabilitation/disability benefits, which were a barrier to feeling independent and in control of their own lives (Holopainen, Vuoskoski, et al. 2022). This reliance on external support sources, coupled with uncertainty about the continuity of rehabilitation benefits, added stress and anxiety, affecting their overall well‐being, and complicating their engagement with physiotherapy and other healthcare services (Holopainen, Vuoskoski, et al. 2022). The lack of reimbursement was a barrier for patients to continue the intervention (Spitaels et al. 2017). Patients who were sick‐listed or unemployed felt ashamed that they needed to be supported by their partners financially, which was not taken into consideration by HCPs, despite the significant impact on patients' well‐being (Holopainen, Vuoskoski, et al. 2022).

### 3.9 Knowledge and Skills

Implementing new interventions required new knowledge and skills, which was time and energy‐consuming for HCPs (Brewer et al. 2021; Faymonville et al. 2021; Holopainen, Piirainen, et al. 2022; S. Peters et al. 2016; van der Vaart et al. 2019). A barrier for HCPs was balancing new tasks with the old (Brewer et al. 2021; Faymonville et al. 2021; Stenberg et al. 2017). Often, HCPs didn't feel educated enough to implement new interventions and requested new skills to accommodate this (Barker et al. 2016; Bouma et al. 2022; Cowell et al. 2018; van der Vaart et al. 2019). One study mentioned that the HCPs seemed unaware of their practice of the new approach when they were confronted by video recording, and they concluded that they did not use the approach as much as they had thought (Fritz et al. 2019). Studies have reported challenges when patients have a negative and biomedical understanding of their condition; even though they indicated they understood the approach, they did not appear to adopt the knowledge into their thoughts and behavioural choices (Bunzli et al. 2016; Fritz et al. 2019; Richmond et al. 2018). Several studies demonstrated that booster training or the ability to revisit online training for HCPs enabled them to keep the new intervention in mind and continuously advance their learning (Faymonville et al. 2021; Holopainen, Piirainen, et al. 2022; Richmond et al. 2018; van der Vaart et al. 2019). A barrier emerged when HCPs experienced a large gap between the training moment and their first appropriate patient (van der Vaart et al. 2019).

#### 3.9.1 Subtheme 1—From ‘Expert’ to ‘Enabler’

Studies emphasise that HCPs have broadened their professional role from being an ‘expert’ to serving as an ‘enabler’, by obtaining new knowledge and skills, and facilitating closer engagement with patients (Brewer et al. 2021; Holopainen, Piirainen, et al. 2022; Littlewood et al. 2015; Synnott et al. 2015). A lack of therapeutic alliance was seen as a barrier to implementing the new approach as it embarrassed some HCPs when discussing patients' thoughts and emotions (Fritz et al. 2019). From both patients' and HCPs' perspectives, several studies highlight the therapeutic alliance as a crucial factor for successful implementation (Bouma et al. 2022; Bunzli et al. 2016; Cowell et al. 2018; Faymonville et al. 2021; Spitaels et al. 2017). A confident relationship was mentioned as a facilitator for a comfortable and confident communication platform (Bunzli et al. 2016).

### 3.10 What Patients Want

Overall, the implementation of the intervention was facilitated, when patients experienced being seen, heard, and understood, being ‘*normal*’ again and confident in their ability to manage their condition (Bøgdal et al. 2021; Bunzli et al. 2016; Holopainen, Vuoskoski, et al. 2022; Spitaels et al. 2017). For example, when patients are worried about severe medical conditions, they want reassurance or a barrier arises (Holopainen, Vuoskoski, et al. 2022). They spoke positively about the intervention when HCPs were specialised, ‘*a nice person*’, helpful, qualified, empathic, supportive, and easy to talk to (Bøgdal et al. 2021; Holopainen, Vuoskoski, et al. 2022; Spitaels et al. 2017). They wanted consistency in which HCP they met and transparency in the intervention to feel comfortable and to be reimbursed (Bøgdal et al. 2021; Spitaels et al. 2017). A barrier arose when patients were confused or frustrated when having various explanations and advices from different HCPs (Holopainen, Vuoskoski, et al. 2022). They wanted not to be defined by their pain condition and not limited participation in their daily life, not to be ashamed of their condition, and not to worry about their future prognosis (Bunzli et al. 2016; Holopainen, Vuoskoski, et al. 2022). HCPs and patients highlighted that the implementation was facilitated when interventions were flexible and individualised to fit into patients' lives, such as jobs and small children (Brewer et al. 2021; Cowell et al. 2018; Faymonville et al. 2021; Fritz et al. 2019; Holopainen, Piirainen, et al. 2022; S. Peters et al. 2016; Spitaels et al. 2017; van der Vaart et al. 2019).

### 3.11 Identified TDF Barriers and Facilitators

**FIGURE 3:**
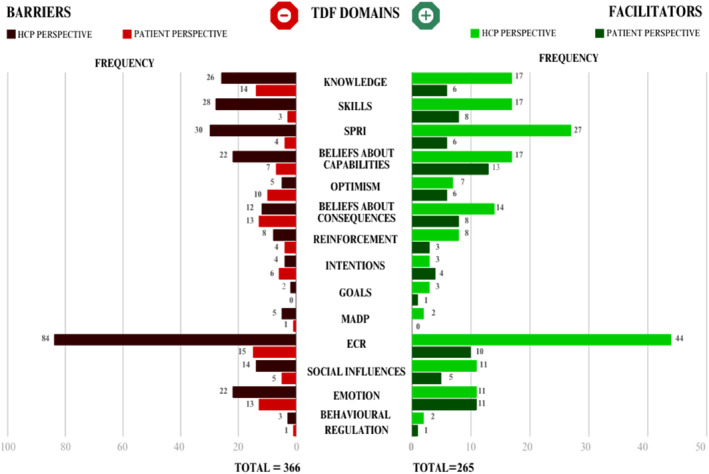
Identified TDF barriers and facilitators.

When applying the TDF, the 229 codes were given 626 new codes: 366 codes to the barriers and 265 codes to the facilitators (Figure 3). The barriers were coded to all the 14 TDF domains for both the HCP's and the patient's perspective and most frequently, to the TDF domains Environmental Context and Resources (ECR) (HCP *n* = 84, 23.5%, and patient *n* = 15, 4.2%), Knowledge (HCP *n* = 26, 7.2% and patient *n* = 14, 3.9%), and Emotion (HCP *n* = 22, 6.1% and patient *n* = 13, 3.6%). Likewise, the facilitators were most frequently coded to the TDF domains ECR within both perspectives (HCP *n* = 44, 16.6%, and patient *n* = 10, 3.8%) and Belief about Capabilities (HCP *n* = 17, 6.4%, and patient *n* = 13, 4.9%). Facilitators and barriers just within the HCP perspective were frequently coded to Social/professional role and identity (SPRI) (Barriers *n* = 30, 8.3%, facilitators *n* = 27, 10.2%) and Skills (barriers *n* = 28, 7.8%, facilitators *n* = 17, 6.4%). Facilitators within the patients' perspective were frequently coded to Emotions (*n* = 11, 4.2%) and barriers to beliefs about consequences (*n* = 13, 3.6%). The facilitators covered all 14 TDF domains, including both HCP and patients' perspectives. We did not find facilitators relating to the domain Memory, Attention and Decision Processes (MADP) within the HCPs' perspective, and no facilitators relating to the domain Goals within the patients' perspective. Within ‘Knowledge’ and ‘Skills’, a significant barrier for HCPs was a limited understanding of the intervention's components, including its procedures and the specific skills required (Barker et al. 2016; Bouma et al. 2022; Cowell et al. 2018; Fritz et al. 2019; van der Vaart et al. 2019). This gap in knowledge and skills hindered effective implementation, as HCPs were uncertain about the expectations and lacked confidence in their ability to carry out the intervention without additional skills development (Barker et al. 2016; Faymonville et al. 2021; Holopainen, Piirainen, et al. 2022; Richmond et al. 2018; Synnott et al. 2016). Patients reported that a lack of both knowledge and skills, creating uncertainty and dependence related to their condition and HCP, posing as a barrier for the acceptance of new treatments (Bunzli et al. 2016; Fritz et al. 2019; Holopainen, Vuoskoski, et al. 2022; Richmond et al. 2018). Furthermore, adequate skills and knowledge, gave patients tools to effectively manage their pain and the uncertainty associated with sudden flare‐ups or changes in their condition (Bunzli et al. 2016; Fritz et al. 2019; Holopainen, Vuoskoski, et al. 2022; Richmond et al. 2018). Related to the ‘Memory, Attention, and Decision Processes’ and ‘Behavioural Regulation’ domains, prior memories with similar interventions also influenced the implementation and uptake of new interventions in clinical practice (Bouma et al. 2022; Fritz et al. 2019; Holopainen, Vuoskoski, et al. 2022; Lentz et al. 2023; Spitaels et al. 2017; Stenberg et al. 2017; van der Vaart et al. 2019; Waddington et al. 2017). For some HCPs and patients, previous positive experiences and memories with comparable interventions or providers served as a facilitator, increasing confidence and willingness to engage with new interventions (Holopainen, Piirainen, et al. 2022; Holopainen, Vuoskoski, et al. 2022; Synnott et al. 2016). Conversely, negative memories acted as a barrier, as HCPs and patients feared repeating past failures, potentially leading to a poorer outcome for the patient (Fritz et al. 2019; Holopainen, Vuoskoski, et al. 2022; Lentz et al. 2023). For the ‘Beliefs about Capabilities’ and ‘Beliefs about Consequences’ barriers concerned a lack of self‐confidence related to individual abilities and negative outcome expectancies based on the lack of capabilities of the surrounding persons involved in the process (Kaloty et al. 2023; Lentz et al. 2023). Other factors such as openness to new interventions and its outcome acted as facilitators for both patients and HCPs (Kaloty et al. 2023; Richmond et al. 2018; Synnott et al. 2016). Factors coded from ‘Optimism’ illustrate comparable findings in terms of optimism about the potential benefits of new interventions that can facilitate implementation, while pessimism may hinder it (Bouma et al. 2022; Cuperus et al. 2013; Faymonville et al. 2021; Fritz et al. 2019; Holopainen, Vuoskoski, et al. 2022; Richmond et al. 2018; Spitaels et al. 2017; Waddington et al. 2017). In addition, factors from ‘Emotion’ may overall influence the emotional response to the adoption of the new intervention, whether positive or negative (Bouma et al. 2022; Bunzli et al. 2016; Holopainen, Vuoskoski, et al. 2022; Waddington et al. 2017). For example, anxiety about the effectiveness, fear of negative outcome failure, and stress related to changing practices (Bouma et al. 2022; Bunzli et al. 2016; Holopainen, Vuoskoski, et al. 2022; Waddington et al. 2017). Barriers coded to the SPRI domain, included internal insecurity related to both professional and personal identity (Barker et al. 2016; Faymonville et al. 2021; Kaloty et al. 2023; Richmond et al. 2018), lacking organisational commitment (Holopainen, Piirainen, et al. 2022; Richmond et al. 2018; Waddington et al. 2017), and absence of clinical leadership (Holopainen, Piirainen, et al. 2022; S. Peters et al. 2016; Stenberg et al. 2017). Conversely, leadership was considered a key facilitator for HCPs related to the implementation and continuation of new interventions (Bouma et al. 2022; Brewer et al. 2021; Faymonville et al. 2021; Fritz et al. 2019; Stenberg et al. 2017; Synnott et al. 2016; van der Vaart et al. 2019). Furthermore, positive previous knowledge being transferred onto new interventions was reported as a facilitator for implementation (Kaloty et al. 2023). Codes related to ‘Goals’ only showed facilitators surrounding the importance of a detailed action planning by providing clear steps and timelines (Bouma et al. 2022; Brewer et al. 2021; Faymonville et al. 2021; Fritz et al. 2019; Holopainen, Piirainen, et al. 2022; Holopainen, Vuoskoski, et al. 2022; Spitaels et al. 2017; Stenberg et al. 2017; van der Vaart et al. 2019; Waddington et al. 2017). Relating to the ‘Environmental Context and Resources’ domains a recurring facilitator was support from leaders and colleagues, which was essential for successful implementation from the perspectives of both healthcare professionals (HCPs) and patients (Bouma et al. 2022; Brewer et al. 2021; Faymonville et al. 2021; Fritz et al. 2019; Holopainen, Piirainen, et al. 2022; Holopainen, Vuoskoski, et al. 2022; Spitaels et al. 2017; Stenberg et al. 2017; van der Vaart et al. 2019; Waddington et al. 2017). Effective communication and the presence of strong social networks within treatment teams promoted internal referrals and ensured interventions remained a priority during implementation (Cowell et al. 2018; Holopainen, Piirainen, et al. 2022; van der Vaart et al. 2019). For example, team meetings facilitate the integration of interventions into daily practice, improving their visibility and uptake (). Similarly, the leadership of clinical champions played a pivotal role in driving change by inspiring confidence and taking on additional responsibilities outside their normal work roles (Bouma et al. 2022; Lentz et al. 2023; Stenberg et al. 2017; Waddington et al. 2017). Conversely, barriers such as organisational constraints were frequently reported. Internal developments, such as re‐organisations, hindered the continuity of trained therapists, while heavy workloads left little time for HCPs to familiarise themselves with new programs or attend training sessions (Cowell et al. 2019; Holopainen, Vuoskoski, et al. 2022). Other factors were related to limited the reimbursement models, which further acted as a barrier by restricting coordinated care. The ‘Social Influences’ domains frequently emerged as both a barrier and facilitator. Supportive relationships within the workplace were key facilitators for HCPs and patients. Positive communication between colleagues encouraged shared learning and reduced resistance to change, while patients often described gaining trust and confidence in their HCPs as instrumental in maintaining their treatment adherence. However, some HCPs reported feeling isolated within their work communities, encountering scepticism from colleagues, or struggling with insecurity about their skills in implementing novel approaches (Bunzli et al. 2016; Kaloty et al. 2023). For patients, some felt that their practitioner no longer met their expectations, particularly when it transitioned away from their preferred treatment (Holopainen, Vuoskoski, et al. 2022). This increased scepticism regarding the skills‐level of the HCP and delayed engagement in the treatment plan (Holopainen, Vuoskoski, et al. 2022). For an overview of the TDF coding, see Supporting Information S1: Appendix 3.

## 4 Discussion

While the research on the barriers and facilitators for the implementation of interventions for managing chronic MSK pain is limited, this review unveils insights into what may either enable or challenge the implementation of interventions across any healthcare setting. Most of the barriers and facilitators were experienced by the HCPs. Furthermore, our study revealed that the barriers often were related to either Environmental Context and Resources (e.g., limited availability of resources and financial constraints) or knowledge (e.g., gaps in understanding the intervention). With regard to facilitators, most were reported as either Belief about Capabilities (e.g., confidence in the intervention's effectiveness) or Environmental Context and Resources (e.g., availability of supportive structures). These insights may help explain the importance of targeted interventions, addressing TDF domain barriers while leveraging facilitators to drive meaningful change and practice adoption within healthcare. Our findings indicate that patients' need for recognition and understanding is consistent across different healthcare sectors, aligning with previous research (Koesling and Bozzaro 2021; Toye et al. 2021). Shared decision‐making has proven effective in meeting these expectations and enhancing patient outcomes (Stacey et al. 2024; Tousignant‐Laflamme et al. 2017), emphasising the importance of considering individual preferences in implementing interventions. The implementation process is also influenced by individual factors, such as being a nice and empathic HCP (Holopainen, Vuoskoski, et al. 2022). While practicing empathy is feasible and an important skill (Linton et al. 2017; Toye et al. 2021), achieving sustainable implementation remains challenging (Simpson et al. 2021). Consistently, with other literature, our study identified conflicting information from different HCPs as a barrier to implementation (Dragsbæk et al. 2023). Multiple and different explanations call for more openness at the organisational level when implementing a new intervention (Briggs et al. 2019; Koesling and Bozzaro 2021). Additionally, our study identified the lack of prioritisation and high status for managing musculoskeletal pain as a barrier, consistent with existing research categorising it as a low‐ranking illness (Lehti et al. 2017). Various reasons may contribute to the low status of working with chronic MSK pain patients, including lack of support and financial resources at organisational and political levels. This can lead to HCPs prioritising financial gain over patient well‐being (Fritz et al. 2019; Saini et al. 2017). Previous reviews have focused on barriers and facilitators for implementation, but they have been limited to single groups of HCPs (physiotherapists (Gardner et al. 2017; Holopainen et al. 2020; Synnott et al. 2015), or general practitioners (Parsons et al. 2007)), single pain conditions (Low back pain (Gardner et al. 2017; Synnott et al. 2015) or osteoarthritis (Egerton et al. 2017)) or specific sectors such as primary care (Ng et al. 2021). Our scoping review, however, takes a broader approach, examining barriers and facilitators across any healthcare sectors for both HCPs and patients with chronic MSK pain. Many similarities were found with existing literature from various systematic reviews (Gardner et al. 2017; Holopainen et al. 2020; Ng et al. 2021; Parsons et al. 2007; Synnott et al. 2015). Our study highlights that factors affecting implementation for HCPs often revolve around interpersonal skills and professionalism. Good interpersonal skills are perceived to facilitate implementation, whereas addressing sensitive topics can be perceived as a barrier due to fear of opening a ‘Pandora's box’, as suggested in previous studies (Holopainen et al. 2020; Ng et al. 2021). Our review reaffirms the notion that asking patients about sensitive topics may be considered beyond HCP's professionalism (Gardner et al. 2017; Synnott et al. 2015). Additionally, Dong et al. suggest that discussing sensitive topics in cancer patients is ideal, despite HCPs feeling inadequate in their competencies (Dong et al. 2016). Moreover, time constraints during consultations may lead to the neglect of addressing sensitive topics, as highlighted in previous research (Keeley et al. 2014). Ultimately, these findings underscore the complexity of implementing interventions in healthcare settings.

### 4.1 Strengths and Limitations

This study has several strengths and limitations, which should be considered by readers. A major strength of this study is the extensive literature search developed in collaboration with an experienced research librarian and conducted in multiple databases. Because of the large number of articles, it is plausible that our search provides a satisfactory overview of the current peer‐reviewed papers related to the area. Importantly, we did not include non‐peer‐reviewed papers, which may have given us more insights into the barriers and facilitators within the field. Most of the studies included originated from European countries and hence, it might limit the applicability of our findings to non‐western countries, and low‐ and middle‐income countries. This highlights the need for more research within other settings and geographical locations. Only one study exclusively used virtual care to deliver their intervention. This study is less represented in the results due to the context‐specific barriers and facilitators and their focus on evaluating the specific intervention. It is considerable how exclusion criteria on studies with virtual care would have resulted in more homogeneity of the included studies. Hence, this study's scope explores a broader perspective and the eligibility criteria are less restrictive. All members of the investigating team were physiotherapists, which should be recognised when interpreting the results of this study.

### 4.2 Implications

Recognising the complex interplay between contextual factors and individual perspectives is essential in defining barriers and facilitators to effectively implement interventions. Our study underscores the importance of tailoring implementation strategies to address these factors comprehensively. Drawing from successful examples in the cancer field, organisational and political support emerges as an enabler for implementation success (Dahl et al. 2017). Moving forward, future research endeavours should delve deeper into understanding the multifaceted dynamics surrounding the implementation of interventions for chronic MSK pain. This necessitates exploration beyond individual‐level factors to encompass organisational and political influences. Additionally, efforts should be directed towards addressing the gap between current interventions and the expectations from both HCPs and patients, particularly regarding the disparity between routine care and intervention approaches as uncovered in the theme ‘long way from usual care’. Furthermore, tackling the issue of low‐value care, characterised by underutilisation of high‐value care and overuse of low‐value care, presents a significant challenge (Briggs et al. 2019; Foster et al. 2018). This substantiated by low‐value care is continuously easier to access and well‐reimbursed (Bouma et al. 2022; Briggs et al. 2019; Lentz et al. 2023). Strategies such as Choosing Wisely have shown promise in mitigating this issue, but further refinement is necessary to address the underlying complexities (Foster et al. 2018; Rosenberg et al. 2015). Our findings suggest organisational factors, including leadership, culture, and resource allocation, are pivotal in shaping implementation outcomes. Similarly, political mechanisms, such as funding mechanisms and regulatory environments exert significant influence on healthcare practices. By integrating insights from frameworks, researchers, HCPs, and patients can tailor intervention strategies to enhance the effectiveness and sustainability of healthcare initiatives (Birken et al. 2017). This collaborative approach will be instrumental in bridging the gap between research and practice, ultimately enhancing the delivery of healthcare initiatives.

## 5 Conclusion

This review identified barriers and facilitators to implementing interventions for managing patients with chronic MSK pain across multiple healthcare settings. Based on the insights achieved from our scoping review, barriers and facilitators seem to be context‐specific, and take place across several levels (individual, organisational, and political). The identification of barriers such as time constraints, low reimbursement rates, and lack of support (from colleagues, leaders, social network etc.) illustrates the complex interactions of factors influencing the implementation of interventions. Barriers related to the TDF domain of Environmental Context and Resources and Knowledge were recognised as the most prominent. Moreover, the leading facilitators for implementing interventions in clinical practice were related to the TDF domains Belief about Capabilities or Environmental Context and Resources. By acknowledging and addressing these multifaceted barriers and facilitators, HCPs, organisations, and policymakers can work towards enhancing the quality and accessibility of interventions for patients with chronic MSK pain.

## Author Contributions

**Mette Dahl Klausen:** conceptualised, data curation, formal analysis, investigation, methodology, validation, visualisation, writing – original draft. **Line Lindberg and Mette Dahl Klausen:** conceptualised, data curation, formal analysis, investigation, methodology, validation, visualisation, writing – original draft. **Simon Kristoffer Johansen:** conceptualised, formal analysis, methodology, supervision, validation, writing – review and editing. **Michael Skovdal Rathleff:** conceptualised, methodology, project administration, resources, software, supervision, validation, writing – review and editing. **Kristian Damgaard Lyng:** conceptualised, data curation, formal analysis, investigation, methodology, project administration, resources, software, supervision, validation, visualisation, writing – original draft.

## Conflicts of Interest

The authors declare no conflicts of interest.

## Supporting information

Supporting Information S1

## Data Availability

The data that support the findings of this study are available from the corresponding author upon reasonable request.
